# The excess body fat is a potential mediator between consumption of ultra-processed foods and cardiometabolic risk in normal-weight Brazilian children

**DOI:** 10.3389/fnut.2025.1726016

**Published:** 2026-01-07

**Authors:** Bruna Clemente Cota, Leidjaira Lopes Juvanhol, Mariana de Santis Filgueiras, Patrícia Feliciano Pereira, Juliana Farias de Novaes

**Affiliations:** 1 Department of Nutrition and Health, Universidade Federal de Viçosa (UFV), Campus Universitário, Viçosa, Minas Gerais, Brazil; 2 Instituto de Saúde Coletiva, Universidade Federal do Estado do Rio de Janeiro (Unirio), Rio de Janeiro, Brazil

**Keywords:** food consumption, adiposity, metabolic syndrome, pediatrics, nutritional epidemiology

## Abstract

**Background/objective:**

Studies indicate an association between the consumption of ultra-processed foods (UPF) and cardiometabolic risk; however, this relationship is still unclear in normal-weight children, and it is not yet known whether the Normal-Weight Obesity (NWO) phenotype may be a mediator between this relationship. We aimed to evaluate the mediating role of the NWO phenotype in the association of UPF consumption with cardiometabolic risk factors.

**Methods:**

This cross-sectional study was carried out with 242 normal-weight children aged 8 to 9 y in Viçosa, Minas Gerais, Brazil. Cardiometabolic risk was assessed as a latent variable using the following indicators: homeostatic model assessment for insulin resistance (HOMA-IR), leptin, mean arterial pressure (MAP), and waist circumference (WC). Three 24-h dietary recalls were performed to evaluate NOVA classification groups. The NWO phenotype was defined as normal-weight according to body mass index and high body fat assessed by dual-energy x-ray absorptiometry. A structural equation model was used to test direct and indirect associations.

**Results:**

We found a direct association of UPF consumption [SC (standardized coefficient): −0.193, *p* = 0.02] and the NWO phenotype (SC: 0.819, *p* < 0.001) with cardiometabolic risk; as well as between UPF consumption and the NWO phenotype (SC: 0.212, *p* = 0.03). In addition, an indirect association was observed between UPF consumption and cardiometabolic risk, being mediated by the NWO phenotype (SC: 0.174, *p* = 0.04).

**Conclusion:**

Our findings indicate the potential influence of excess body fat on the pathway between UPF consumption and cardiometabolic risk in children with adequate BMI/age.

## 1 Introduction

The presence of cardiometabolic risk factors in childhood is associated with an increased likelihood of developing metabolic and cardiovascular diseases – such as hypertension and type 2 diabetes – in adulthood (1, 2). The increase in prevalence of these diseases represents a major impact on society, overloading health systems and causing high costs (3). Although most studies report the effect of childhood obesity (classified based on BMI/age) on cardiometabolic complications, it is noteworthy that normal-weight children can also present these complications, including excess body fat, which is called Normal-Weight Obesity (NWO) phenotype (4).

Evidence suggests an increase in the prevalence of this phenotype and that more than one-quarter of children with excess adiposity remain undetected when obesity is diagnosed solely based on BMI (5–7). Therefore, current BMI-based measures may underdiagnose the NWO phenotype and hinder clinically sound approaches to health care and policy (8).

Behavioral factors such as unhealthy eating habits are associated with the presence of cardiometabolic risk factors. Studies indicate an increase in the consumption of ultra-processed foods (UPF) in children and adolescents (9–11). The latest report from the National Study of Infant Feeding and Nutrition (ENANI-2019) revealed a prevalence of UPF consumption among Brazilian children aged 24 to 59 months of 93.0% (12). The consumption of UPF − which are pro-inflammatory (13), rich in additives, sugar, unhealthy fat and sodium (14) − was associated with adiposity, dyslipidemia and other cardiometabolic complications in pediatric population (10, 13, 15, 16). Among children with normal-weight, we observed that the prevalence of the NWO phenotype was higher in those with higher UPF consumption (13).

Despite the existence of studies on the relationship of high UPF consumption with obesity and other cardiometabolic risk factors, this relationship is still unclear in children with normal-weight. Furthermore, few studies have evaluated the pathways linking dietary intake, adiposity and other cardiometabolic risk factors (17, 18); and, according to our knowledge, we did not identify studies that evaluated this interrelationship in childhood, or that considered the clustering of cardiometabolic risk variables.

Therefore, considering that normal-weight children can present unhealthier food consumption and increased adiposity, this study aimed to evaluate the mediating role of the NWO phenotype in the association between UPF consumption and cardiometabolic risk factors in Brazilian children with adequate BMI/age.

## 2 Methods

### 2.1 Participants and study design

This cross-sectional investigation is part of the “Schoolchildren Health Assessment Survey” (PASE, in Portuguese) and included a representative group of 8- and 9-year-old students attending both public and private schools in the urban area of Viçosa, Minas Gerais, Brazil. Viçosa is a city of medium size, with around 72,000 residents, mostly living in urban zones, and is located 227 km southeast of Belo Horizonte, the state capital. Data were collected between May and December 2015.

Study design have been described previously (19, 20). Briefly, the sample size was determined using the Epi Info software (version 7.2; Atlanta, GA), using the formula recommended for cross-sectional designs. This calculation took into account the total population of 8- and 9-year-old schoolchildren of Viçosa in 2015 (*n* = 1,464) (21), expected prevalence of 50% (22), tolerated error of 5, and 95% confidence interval, which resulted in a required sample of 305 participants. The PASE study comprised 378 children through random selection. All data collection procedures were carried out at the Federal University of Viçosa (UFV, in Portuguese) by trained professionals.

Children were not included in the study if they had health conditions that could alter nutritional status or body composition; were under chronic medication affecting glucose and/or lipid metabolism; or if their guardians could not be contacted after three contact attempts. Previous health problems and medication use were assessed by asking the children’s guardians.

In this study, children with thinness (*n* = 12), overweight (*n* = 65) and obesity (*n* = 59) were excluded. Participants with normal-weight and adequate body fat percentage (*n* = 176) were classified as Normal-Weight Lean (NWL); and normal-weight and high body fat (*n* = 66) were classified as NWO phenotype.

The PASE study followed the principles outlined in the Declaration of Helsinki and received approval from the Human Research Ethics Committee of the Federal University of Viçosa (protocol no. 663.171/2014). The participation in the study required that parents or legal guardians of the children sign an informed consent form.

### 2.2 Cardiometabolic risk

The cardiometabolic risk was assessed by four variables: homeostatic model assessment for insulin resistance (HOMA-IR), serum leptin, mean arterial pressure (MAP), and waist circumference (WC).

After a 12-h overnight fast, blood samples were obtained by venipuncture from the antecubital vein at the Clinical Analysis Sector of the UFV Health Center. Fasting insulin concentrations were assessed using the chemiluminescence immunoassay method at the Diagnósticos do Brasil Laboratory and quantified with the Elecsys Insulin® test (Roche Diagnostics, Indianapolis, IN, United States). The HOMA-IR index was computed using the formula: fasting insulin (μU/mL) × fasting glucose (mmol/L)/22.5 (23). Serum leptin levels were analyzed in duplicate through the enzyme immunoassay technique, with intra-assay and inter-assay coefficients of variation below 13.3 and 12.7%, respectively (KAP2281, DIAsource®, Louvain-la-Neuve, Belgium; standardized by Diagnósticos do Brasil).

Blood pressure was assessed in triplicate with digital monitors (Omron®, Vernon Hills, IL, United States) with the children seated after a minimum rest of 5 min. Mean arterial pressure (MAP) was estimated using the formula: MAP = ⅓ (systolic blood pressure) + ⅔ (diastolic blood pressure), based on the average of systolic and diastolic readings in mmHg (24).

The anthropometric measures were evaluated by trained researchers, including the waist circumference (WC) that was measured with a non-elastic tape, positioning it at the midpoint between the lower edge of the ribs and the upper limit of the iliac crest (25).

### 2.3 UPF consumption

Dietary intake was evaluated using the mean of three 24-h dietary recalls (24HR) applied on non-consecutive days, one of which corresponded to a weekend. The recalls were answered jointly by the child and their guardian. Trained dietitians conducted the 24HR following the 5-step multiple-pass approach (26). To improve portion size estimation and enhance data accuracy, interviewers used household measuring utensils and photographic food records (27) during the interviews.

Portion sizes were converted into grams (g) and milligrams (mg) to calculate energy intake (kcal). Dietary composition was analyzed using the software Diet Pro® 5i, version 5.8. For this purpose, the Brazilian Food Composition Table (TACO, in Portuguese) (28) was used as the primary reference, complemented by the USDA Food Composition Database (29) when necessary. Foods were then categorized by level of processing, following the NOVA classification system (30, 31). Industrial formulations that combine ingredients intended exclusively for industrial use (such as additives, antioxidants, stabilizers and preservatives), designed to create ready-to-eat products that replace traditional culinary preparations, have been identified as ultra-processed foods (UPF), such as soft drinks, fast food, cookies and snacks (30, 31) (Supplementary material). UPF consumption was assessed by the proportion that these foods represented in the total energy consumed. Subsequently, this variable was categorized into tertiles.

### 2.4 Normal-weight obesity (NWO) phenotype – mediating variable

The NWO phenotype was defined in children who presented normal weight based on BMI-for-age while simultaneously exhibiting elevated body fat levels (13). The weight and height were measured using standardized procedures (32), using, respectively, an electronic digital scale (Tanita® Ironman Model BC 553, Tanita Corporation of America Inc., Arlington Heights, United States) with a 150 kg capacity and 100 g precision, and a vertical stadiometer (Alturexata®, Belo Horizonte, Brazil) in centimeters and millimeters. BMI-for-age z-scores were calculated as weight (kg) divided by the square of height (m^2^), considering the sex to classify the nutritional status through WHO Anthro Plus software (33).

The body fat percentage (%) was measured by a trained technician using Dual Energy X-ray Absorptiometry (DXA) (Lunar Prodigy Advance, GE Medical Systems Lunar, Milwaukee, WI, United States). During the scan, children lay in a supine position wearing light clothing without metal accessories. High body fat was defined as ≥25% for girls and ≥20% for boys (34), as used by other studies (13, 35–38).

### 2.5 Confounding variables

Information on child’s sex (female and male), skin color (white/non-white), age (years), per capita family income (USD) and practice of some physical activity outside of school (yes/no) were filled out in a semi-structured questionnaire during interview with child and its guardian.

### 2.6 Data analysis

*Exposure*: UPF consumption (categorized in tertile).

*Mediator*: NWO.

*Outcome*: Latent variable of cardiometabolic risk (components: HOMA-IR, leptin, MAP and WC).

*Covariates*: Sex, skin color, age, per capita income and practice of some physical activity outside of school (yes/no).

*Statistical analyses*: Descriptive analysis was carried out in the software Stata® version 14 (StataCorp LP, College Station, TX, United States). The consistency and distribution of numerical variables were evaluated by histograms, and skewness and kurtosis coefficients; and the Shapiro–Wilk was used for the normality test. Categorical variables were reported as absolute and relative frequencies, while numerical variables were presented as mean ± standard deviation (SD) or median with interquartile range (IQR). Comparisons of numerical variables were performed using the Student’s *t*-test or the nonparametric Mann–Whitney test, and categorical variables were analyzed using Pearson’s chi-square test or the chi-square test for linear trend.

**Figure 1:**
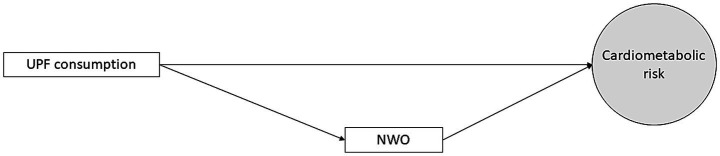
Theoretical structural equation model hypothesized for the mediating role of the NWO phenotype on the relationship between UPF consumption and cardiometabolic risk (Viçosa, MG, Brazil, 2015). UPF, ultra-processed foods; NWO, normal-weight obesity.

The structural equation modeling (SEM) was performed using the Mplus software, version 8.6 (Muthén & Muthén, Los Angeles, CA, United States). The hypothesized structural equation model was presented in Figure 1, in which the associations of UPF consumption with child’s cardiometabolic risk were investigated, considering the NWO phenotype as mediated variable. The weighted least squares mean, and variance adjusted (WLSMV) estimator was used for parameter estimation. Furthermore, the theta parameterization was used to control the differences in residual variances (39). The standardized coefficients (SC) were estimated with their respective *p* values. The database had four missing values for the cardiometabolic risk variables, but Mplus used all available information to estimate the model parameters.

Model fit was assessed using the Tucker-Lewis Index (TLI) and the Comparative Fit Index (CFI), where values above 0.9 indicate good model fit (40); and Root Mean Square Error of Approximation (RMSEA) values less than or equal to 0.08 (41, 42). The standardized factor loadings for the latent variables were considered acceptable when greater than 0.30 and statistically significant (*p* < 0.05) (40).

The significance level was 5% for all hypothesis tests.

## 3 Results

### 3.1 Sample characterization

This study included 242 children with adequate BMI/age, of which 51.6% are female. The prevalence of the NWO phenotype was 27.3%, being higher in non-white children. Furthermore, those with the NWO phenotype had higher per capita income, insulin resistance and waist circumference than NWL children (Table 1). The mean UPF consumption (% caloric contribution to total caloric intake) in the sample was 44.62 (14.53). Among children with the NWO phenotype, the majority were categorized in the second and third tertiles of UPF consumption. On the other hand, NWL children consumed less UPF (mostly in the first tertile) (Table 1).

**Table 1:** Sample characterization (Viçosa, MG, Brazil, 2015).

Characteristics	Overall (*n* = 242)	NWL (*n* = 176)	NWO (*n* = 66)	*p*-value
	*n* (%)	*n* (%)	*n* (%)	
Sex				
Female	125 (51.65)	89 (71.20)	36 (28.80)	0.58^†^
Male	117 (48.35)	87 (74.36)	30 (25.64)	
Skin color				
White	164 (67.77)	126 (76.83)	38 (23.17)	**0.04** ^†^
Non-white	78 (32.23)	50 (64.10)	28 (35.90)	
Age (years)^1^	8.50 (0.50)	8.50 (0.50)	8.50 (0.50)	0.57
Per capita family income (USD/month)^1^	139.95 (91.89; 225.23)	120.1 (79.6;200.1)	156.7 (120.1;329.1)	**<0.001**
Practice of some physical activity outside of school				0.46^†^
Yes	75 (30.99)	51 (68.00)	24 (32.00)	
No	166 (68.60)	124 (74.70)	42 (25.30)	
HOMA-IR^1^	0.79 (0.56; 1.20)	0.75 (0.53; 1.08)	0.91 (0.66; 1.42)	**0.008**
Leptin (ng/mL)^1^	1.05 (0.10; 3.00)	1.10 (0.10; 2.55)	0.90 (0.30; 5.40)	0.05
MAP (mmHg)^1^	72.17 (68.33; 76.33)	71.67 (68.00; 76.33)	72.67 (69.33; 78.00)	0.11
WC (cm)^1^	55.50 (52.60; 58.00)	54.00 (52.00; 56.70)	58.80 (56.80; 61.00)	**<0.001**
NOVA classification (%TCI tertile)				
UPF [mean (SD)]				**0.030**^**ψ**^
T1 [29.37 (7.44)]	81 (33.47)	67 (82.72)	14 (17.28)	
T2 [44.20 (2.97)]	81 (33.47)	55 (67.90)	26 (32.10)	
T3 [60.49 (9.21)]	80 (33.06)	54 (67.50)	26 (32.50)	
Processed foods [mean (SD)]				0.510^ψ^
T1 [2.53 (1.93)]	81 (33.47)	59 (72.84)	22 (27.16)	
T2 [8.04 (1.58)]	81 (33.47)	55 (67.90)	26 (32.10)	
T3 [16.61 (5.38)]	80 (33.06)	62 (77.50)	18 (22.50)	
Culinary ingredients [mean (SD)]				0.123^ψ^
T1 [0.81 (0.56)]	81 (33.47)	55 (67.90)	26 (32.10)	
T2 [2.52 (0.53)]	81 (33.47)	58 (71.60)	23 (28.40)	
T3 [5.33 (1.69)]	80 (33.06)	63 (78.75)	17 (21.25)	
Unprocessed or minimally processed [mean (SD)]				0.058^ψ^
T1 [29.99 (6.70)]	81 (33.47)	55 (67.90)	26 (32.10)	
T2 [43.83 (2.89)]	81 (33.47)	56 (69.14)	25 (30.86)	
T3 [56.77 (5.80)]	80 (33.06)	65 (81.25)	15 (18.75)	

NWL, normal-weight lean; NWO, normal-weight obesity; HOMA-IR, homeostatic model assessment for insulin resistance; MAP, mean arterial pressure; WC, waist circumference; UPF, ultra-processed food; TCI, total caloric intake; T, tertile.

Student’s *t*-test/Mann–Whitney test.

Bold values (*p* < 0.05).

^†^Pearson’s chi-squared test.

^ψ^Linear trend chi-square.

^1^Mean/median (standard deviation/interquartile range).

### 3.2 Latent variable and model fit

A latent variable for cardiometabolic risk was created. Initially, an exploratory analysis based on theoretical and statistical criteria was performed to select the variables representing cardiometabolic risk. Subsequently, a confirmatory analysis was performed, including only variables with satisfactory fit indices in the latent variable. Other cardiometabolic risk variables in the PASE study were tested, such as triglycerides, HDL cholesterol, uric acid, adiponectin, ferric reducing antioxidant power (FRAP), malonaldehyde (MDA) and superoxide dismutase (SOD), but the latent variable did not show satisfactory adjustment indices (data not shown). These findings justified the use of HOMA-IR, leptin, MAP and WC as components of the latent variable in this study.

The latent variable presented adequate and statistically significant factor loads. In general, the model showed good quality of fit, satisfying most of the criteria: RMSEA: 0.07; CFI and TLI: 0.9. Furthermore, the model presented *χ*^2^ = 49.839, Degrees of Freedom = 23 and *p*-value = 0.001. Factorial loadings were higher for HOMA-IR and waist circumference (Table 2).

**Table 2:** Factor loadings of the observed variables composing the latent variable of cardiometabolic risk (Viçosa, MG, Brazil, 2015).

Observed variables	Standardized factor loadings
HOMA-IR	0.724
Leptin (ng/mL)	0.559
MAP (mmHg)	0.310
WC (cm)	0.580

*p* < 0.05.

HOMA-IR, homeostatic model assessment for insulin resistance; MAP, mean arterial pressure; WC, waist circumference.

### 3.3 Structural equation model

*Direct association*: A positive association was found between the NWO phenotype and cardiometabolic risk (SC = 0.819, *p* < 0.001), and between UPF consumption and the NWO phenotype (SC = 0.212, *p* = 0.03). Contrary to expectations, we found a negative direct association between UPF consumption and cardiometabolic risk (SC = −0.193, *p* = 0.02). It is important to consider that this result may have been affected by other potential mediators not evaluated in this analysis (direct association) (Table 3).

**Table 3:** Standardized coefficients (SC) for the structural equation model (*n* = 242) (Viçosa, MG, Brazil, 2015).

Direct and indirect associations	Standardized coefficients	95%CI	*p*-value
Direct association			
UPF → cardiometabolic risk	**−0.193**	**−0.362; −0.025**	**0.02**
NWO → cardiometabolic risk	**0.819**	**0.696; 0.942**	**<0.001**
Sex → cardiometabolic risk	−0.111	−0.262; 0.040	0.15
Skin color → cardiometabolic risk	0.128	−0.018; 0.273	0.09
Age → cardiometabolic risk	0.133	−0.015; 0.281	0.08
Per capita family income → cardiometabolic risk	−0.122	−0.298; 0.055	0.18
Practice of physical activity → cardiometabolic risk	0.037	−0.107; 0.181	0.61
UPF → NWO	**0.212**	**0.020; 0.404**	**0.03**
Sex → NWO	0.058	−0.117; 0.233	0.52
Skin color → NWO	−0.093	−0.268; 0.082	0.30
Age → NWO	0.072	−0.100; 0.245	0.41
Per capita family income → NWO	0.057	−0.112; 0.225	0.51
Practice of physical activity → NWO	0.029	−0.160; 0.218	0.76
Sex → UPF	−0.097	−0.238; 0.044	0.18
Skin color → UPF	−0.083	−0.231; 0.064	0.27
Age → UPF	−0.043	−0.184; 0.098	0.55
Per capita family income → UPF	0.048	−0.099; 0.194	0.52
Practice of physical activity → UPF	−0.116	−0.275; 0.043	0.15
Indirect association			
UPF → NWO → cardiometabolic risk	**0.174**	**0.009; 0.338**	**0.04**
Total association (direct + indirect)			
UPF → cardiometabolic risk	−0.020	−0.193; 0.153	0.82

UPF, ultra-processed foods; NWO, normal-weight obesity; 95%CI, 95% confidence interval. Structural equation modeling. Model fit information: root-mean-square error of approximation (RMSEA): 0.07; comparative fit index (CFI) and Tucker Lewis index (TLI): 0.9. *χ*^2^ = 49.839, degrees of freedom = 23 and *p*-value = 0.001. Adjusted by child’s sex, skin color, age, per capita family income, and practice of physical activity. Bold values (*p* < 0.05).

*Indirect association (mediating effect)*: We observed a positive indirect association between UPF consumption and cardiometabolic risk, being partially mediated by the NWO phenotype (SC = 0.174, *p* = 0.04). This finding reinforces that the association between high UPF consumption and increased cardiometabolic risk was partially explained by excess body fat in normal-weight children (Table 3).

*Total association (direct + indirect)*: No significant total effect was observed between UPF and cardiometabolic risk (*p* = 0.82) (direct + indirect association) (Table 3).

**Figure 2:**
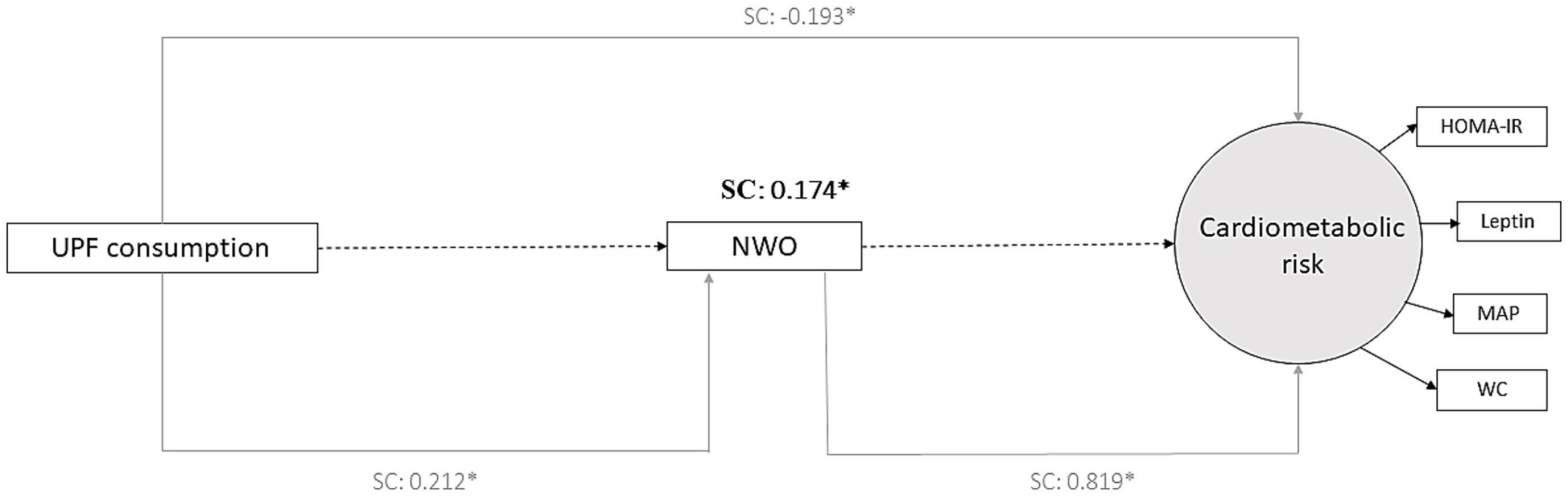
Final model of structural equations modeling (SEM) showing the mediating effect of the NWO phenotype on the relationship between UPF consumption and cardiometabolic risk (Viçosa, MG, Brazil, 2015). SC, standardized coefficient; UPF, ultra-processed foods; NWO, normal-weight obesity; HOMA-IR, homeostatic model assessment for insulin resistance. MAP, mean arterial pressure. WC, waist circumference. *p < 0.05. Values are standardized coefficients. The observed variables are presented in rectangular and the latent variable in circular form. Adjusted by child’s sex, skin color, age, per capita family income and practice of physical activity.

The mediating effect of the NWO phenotype is summarized in Figure 2.

## 4 Discussion

We observed in this study that the association between high UPF consumption and increased cardiometabolic risk was partially explained by excess body fat. Therefore, our study supports the hypothesis that high consumption of UPF may contribute to increased adiposity. Consequently, excess fat can cause hormonal and inflammatory changes, increasing cardiometabolic risk – even in children with normal BMI. However, we emphasize that our cross-sectional study is unable to infer causality. We did not find other studies that investigated this relationship in early ages.

The NWO phenotype can remain unrecognized for years, as it occurs in normal-weight individuals and often at a young age. However, its early detection is crucial, as it is associated with increased cardiometabolic risk (43–45). This situation highlights the limitations of BMI, which does not distinguish between lean, adipose, and bone mass (46). Recently, experts have emphasized that the exclusive use of BMI may underestimate or overestimate adiposity, in addition to providing insufficient information to guide clinical decisions and health policies (8). Therefore, early identification of NWO, as well as its associated factors, is essential for a more accurate health assessment.

An association of high UPF consumption with the NWO phenotype in children was found in this study. The higher UPF consumption, a modifiable risk factor, has been associated with adiposity and other cardiometabolic complications, such as increased BMI, WC, and fasting plasma glucose in children (47), mainly due its unbalanced nutritional composition, rich in sugars, fat, sodium, and calorie density (14). Furthermore, it is noteworthy that UPFs were designed to be highly palatable, appealing and energy-dense, with a combination of flavor-enhancing ingredients to generate a strong rewarding stimulus and influence circuits related to feeding facilitation, especially in children (48). This result reinforces the findings of other studies that indicate that the intake of UPF over time can impact the nutritional status and body composition of children and adolescents, favoring the accumulation of body fat (16) and, consequently, associated comorbidities (49).

On the other hand, we found a contradictory result in our study: the negative direct association between UPF consumption and cardiometabolic risk (SC = −0.193, *p* = 0.02). This may be explained by the fact that the consumption of UPF intake could not represent a dietary pattern and can be influenced by several factors, such as income. The relationship between income and UPF consumption in normal-weight Brazilian children is still unclear in the literature. In our sample, we observed that the median of per capita family income increased with the highest tertile of UPF intake (Kruskal-Wallis test: *p* < 0.05; data not shown). High-income populations have more access to food and health services (50); and the consumption of UPF can occur in the context of a better quality of life, access to medical health services and a healthier overall diet in children. Furthermore, the possibility of measurement bias should be considered, particularly the potential for differential underreporting of ultra-processed food consumption among households with different socioeconomic levels, as well as the lack of adjustment for global dietary patterns. In this way, the UPF consumption alone could not be sufficient to present a positive direct association with cardiometabolic risk in our sample. Other potential mediators that we did not evaluate in this study may explain the contradictory result of the direct association between UPF and cardiometabolic risk. We reinforce that the total effect between UPF and cardiometabolic risk (direct + indirect association) was not observed in our study.

It is important to highlight that there was a positive indirect association (SC = 0.174, *p* = 0.04) between the UPF consumption and the cardiometabolic risk mediated by the NWO phenotype. This association presented almost the same magnitude of the negative direct association (SC = −0.193, *p* = 0.02), reinforcing the influence of excess body fat in this pathway. This finding confirmed our hypotheses that excessive UPF consumption may contribute to increased body fat in normal-weight children (NWO phenotype) and, consequently, be associated with cardiometabolic risk. In this way, we highlight the role of high body fat in the pathway between UPF and cardiometabolic risk in Brazilian normal-weight children. Longitudinal studies are needed to investigate the causal relationships of these variables.

In addition, we found a positive association between the NWO phenotype and cardiometabolic risk, in agreement with findings from previous studies (44, 51). This association can be explained by the fact that, despite normal weight, the excess body fat, especially in the central region, can increase the synthesis and secretion of other molecules, such as adipokines (52). Adipokines act as signaling molecules and have hormonal functions, being involved in the regulation of several systemic processes, such as insulin sensitivity (53, 54). Furthermore, some adipokines, such as leptin in particular, regulate food intake and energy expenditure, promoting satiety and reducing food intake (55, 56). However, leptin is produced proportionally to body fat, and studies have indicated that individuals with obesity have hypothalamic resistance to its action (55, 56). Thus, hyperleptinemia and leptin resistance may be associated with negative health effects, including cardiovascular risk factors such as high blood pressure (57–59).

Among the definitions used for cardiometabolic risk factors in this study, WC and blood pressure stand out, as they are practical, non-invasive, low-cost, and quick measurements. It confirms that even normal-weight children can have cardiometabolic risk factors (44); and the increased WC, MAP, leptin, and insulin resistance may be associated with unhealthier conditions, including obesity (1, 60, 61).

The present study had several strengths. First, definitions of cardiometabolic risk factors have varied widely and we highlight the novelty of this study in using a latent variable, once most other studies investigate the variables separately. Second, body fat was assessed by DXA, a reference method; and three 24-h recalls were used to measure food consumption. Finally, the SEM approach is a robust technique that allowed us to evaluate direct and indirect associations. As a limitation, the cross-sectional design of this study does not allow to stablish a cause-effect relationship. Longitudinal studies are necessary to verify the long-term effect of UPF consumption on children’s health with normal-weight. Second, the pubertal status was not evaluated once the age range of children was limited to 8 and 9 years, and probably homogeneous sexual and hormonal characteristics. Furthermore, we cannot rule out the possibility of residual confounders not assessed in this study, such as parental education, social status, and sleep. Finally, we highlight the inherent limitations of the 24-h recall, such as the possibility of memory bias and the underreporting of ultra-processed and high-energy-density foods; and the potential incorrect classification of foods using the NOVA classification, given the complexity of children’s diets.

We conclude that, even in normal-weight children, the excess body fat was a potential mediator between consumption of ultra-processed foods and cardiometabolic risk in Brazilian children. This study highlights the importance of investigate early the quality of diet and excess of body fat to prevent unhealthier conditions, independent of BMI. The improvement of the modifiable risk factors in the routine of children, such as low diet quality, is necessary even in normal weight individuals and need to be supported by all sectors of society, including the families, schools and health services.

## Data Availability

The datasets are not publicly available due to confidentiality and controlled access policies. Anonymized data may be obtained from the corresponding author upon reasonable request.
