# Supplementary material for: The excess body fat is a potential mediator between consumption of ultra-processed foods and cardiometabolic risk in normal-weight Brazilian children
Source: Front Nutr. 2026 Jan 7;12:1726016. doi: 10.3389/fnut.2025.1726016 (PMC12819215; doi:10.3389/fnut.2025.1726016)
Supplement: Supplementary file 1 [file Table_1.DOCX]

**Supplementary material** − Ultra-processed foods consumed in the PASE-Brazil study

| Ultra-processed foods | Sliced ​​bread  Sweet bread with frosting  Small bread rolls  Fried and/or baked savory snacks  Mortadella  Chocolate milk drink  Dairy drink  Donut  Rice pudding  Chewing gum  Packaged snacks  Boxed juice  Potato sticks  Cookies  Chocolate  Hamburger patty  Sausage  Nuggets  Salami |
| --- | --- |
